# 1886. External Validation of the 4C Mortality Score and the qSOFA for Different Variants of Concerns of SARS-CoV-2 Using Data of the NAPKON Cross-Sectoral Cohort Platform (SUEP)

**DOI:** 10.1093/ofid/ofac492.1513

**Published:** 2022-12-15

**Authors:** Katharina S Appel, Daniel Maier, Sina M Hopff, Lazar Mitrov, Melanie Stecher, Margarete Scherer, Ramsia Geisler, Marina Hagen, Kirsten Haas, Jens-Peter Reese, Steffi Jiru-Hillmann, Olga Miljukov, Carolin E M Jakob, Susana M Nunes de Miranda, Patrick Meybohm, Sabine Hanß, Johanna Erber, Christof Winter, Johannes J Tebbe, Christoph Stellbrink, Yascha Khodamoradi, Julia Schmidt, Frank Hanses, Christian Scheer, Sabine Blaschke, Siri Göpel, Stefan Kluge, Oliver Witzke, Christoph Römmele, Marcin Krawczyk, Andreas Teufel, Jonas Schmid, Daniel Pape, Christian Schütte, Kristin Tausche, Milena Milovanovic, Natalie Krug, Phil-Robin Tepasse, Marlo Verket, Axel Hamprecht, Selcuk Tasci, Martin Hower, Björn-Erik O Jensen, Martin F Sprinzl, Tim Zimmermann, Jörg J Vehreschild

**Affiliations:** Department of Internal Medicine, Hematology/Oncology, Goethe University Frankfurt, Frankfurt am Main, Germany, Frankfurt am Main, Hessen, Germany; University Hospital Frankfurt, Frankfurt am Main, Germany; German Cancer Consortium (DKTK), Heidelberg, Germany, Frankfurt am Main, Hessen, Germany; University of Cologne, Faculty of Medicine and University Hospital Cologne, Department I of Internal Medicine, Center for Integrated Oncology Aachen Bonn Cologne Duesseldorf, Köln, Nordrhein-Westfalen, Germany; University of Cologne, Faculty of Medicine and University Hospital Cologne, Department I of Internal Medicine, Center for Integrated Oncology Aachen Bonn Cologne Duesseldorf, Köln, Nordrhein-Westfalen, Germany; University of Cologne, Faculty of Medicine and University Hospital Cologne, Department I of Internal Medicine, Center for Integrated Oncology Aachen Bonn Cologne Duesseldorf; German Centre for Infection Research (DZIF), Partner Site Bonn-Cologne, Cologne, Germany, Cologne, Nordrhein-Westfalen, Germany; Department of Internal Medicine, Hematology/Oncology, Goethe University Frankfurt, Frankfurt am Main, Germany, Frankfurt am Main, Hessen, Germany; Department of Internal Medicine, Hematology/Oncology, Goethe University Frankfurt, Frankfurt am Main, Germany, Frankfurt am Main, Hessen, Germany; Department of Internal Medicine, Hematology/Oncology, Goethe University Frankfurt, Frankfurt am Main, Germany, Frankfurt am Main, Hessen, Germany; Insitute for Clinical Epidemiology and Biometry, Julius Maximilians Universität Würzburg, Würzburg, Bayern, Germany; Insitute for Clinical Epidemiology and Biometry, Julius Maximilians Universität Würzburg, Würzburg, Bayern, Germany; Insitute for Clinical Epidemiology and Biometry, Julius Maximilians Universität Würzburg, Würzburg, Bayern, Germany; Insitute for Clinical Epidemiology and Biometry, Julius Maximilians Universität Würzburg, Würzburg, Bayern, Germany; University of Cologne, Faculty of Medicine and University Hospital Cologne, Department I of Internal Medicine, Center for Integrated Oncology Aachen Bonn Cologne Duesseldorf; German Centre for Infection Research (DZIF), Partner Site Bonn-Cologne, Cologne, Germany, Cologne, Nordrhein-Westfalen, Germany; University of Cologne, Faculty of Medicine and University Hospital Cologne, Department I of Internal Medicine, Center for Integrated Oncology Aachen Bonn Cologne Duesseldorf, Köln, Nordrhein-Westfalen, Germany; Department of Anaesthesiology, Intensive Care, Emergency and Pain Medicine, University Hospital Würzburg, Würzburg, Würzburg, Bayern, Germany; University Medical Center Göttingen, Department of Medical Informatics, Göttingen, Germany, Göttingen, Niedersachsen, Germany; Technical University of Munich, School of Medicine – University Hospital, Department of Internal Medicine, Gastroenterology, Munich, Bayern, Germany; Technical University of Munich, School of Medicine – University Hospital, Institute for Clinical Chemistry and Pathobiochemistry, Munich, Bayern, Germany; University Medical Center East Westphalia-Lippe, Klinikum Lippe, Department of Gastroenterology and Infectious Disease, Lippe, Nordrhein-Westfalen, Germany; Bielefeld University, Medical School and University Medical Center East Westphalia-Lippe, Klinikum Bielefeld, Academic Department of Cardiology, Bielefeld, Nordrhein-Westfalen, Germany; Department of Internal Medicine, Infectious Diseases, Goethe University Frankfurt, Frankfurt am Main, Germany, Frankfurt am Main, Hessen, Germany; Insitute for Clinical Epidemiology and Biometry, Julius Maximilians Universität Würzburg, Würzburg, Bayern, Germany; Emergency Department and Department for Infection Control and Infectious Diseases, University Hospital Regensburg, Regensburg, Germany, Regensburg, Bayern, Germany; Department of Anaesthesiology, University Medicine Greifswald, Greifswald, Germany, Greifswald, Mecklenburg-Vorpommern, Germany; Emergency Department, University Medical Center Göttingen, FRG, Göttingen, Niedersachsen, Germany; Department of Internal Medicine I, Infectious Diseases, Tübingen University Hospital, Tübingen, Germany; German Centre for Infection Research (DZIF), Clinical Research Unit for healthcare associated infections, Tübingen, Germany, Tübingen, Baden-Wurttemberg, Germany; Department of Intensive Care, University Medical Center Hamburg-Eppendorf, Hamburg, Hamburg, Germany; Department of Infectious Diseases, West German Centre of Infectious Diseases, Universitymedicine Essen, University Duisburg-Essen, Germany, Essen, Nordrhein-Westfalen, Germany; Clinic for Internal Medicine III - Gastroenterology and Infectious Diseases, University Hospital of Augsburg, Augsburg, Germany, Augsburg, Bayern, Germany; Department of Medicine II, Saarland University Medical Center, Saarland University, Homburg, Germany, Homburg, Saarland, Germany; Department of Medicine II, Division of Hepatology, Division of Bioinformatics, Medical Faculty Mannheim, Heidelberg University, Mannheim, Germany; Clinical Cooperation Unit Healthy Metabolism, Center for Preventive Medicine and Digital Health Baden-Württemberg (CPDBW), Medical Faculty Mannheim, Heidelberg University, Mannheim, Germany, Mannheim, Baden-Wurttemberg, Germany; Friedrich-Alexander-Universität Erlangen-Nürnberg, Universitätsklinikum Erlangen, Medicine I, Erlangen, Germany, Erlangen, Bayern, Germany; Department of Internal Medicine I, University Hospital Schleswig Holstein, Campus Kiel, Kiel, Schleswig-Holstein, Germany; Dept. of Medicine I, St. Josef-Hospital, Ruhr-University of Bochum Medical School, Bochum, Nordrhein-Westfalen, Germany; Department of Internal Medicine I, Pulmonology, Carl- Gustav-Carus University Dresden, Germany, Dresden, Sachsen, Germany; Medical Clinic 1, Malteser Krankenhaus St. Franziskus Hospital, Flensburg, Germany, Flensburg, Schleswig-Holstein, Germany; Department of Anesthesiology and Intensive Care, University Hospital Leipzig, Germany, Leipzig, Sachsen, Germany; Department of Medicine B for Gastroenterology, Hepatology, Endocrinology, Clinical Infectiology, Münster, Germany, Münster, Nordrhein-Westfalen, Germany; Department of Medicine I, Clinical Study Center, University Hospital Aachen, Aachen, Nordrhein-Westfalen, Germany; University Medical Clinic of Medical Microbiology and Virology, Department of Human Medicine, University Oldenburg, Germany, Oldenburg, Niedersachsen, Germany; Department of Pulmonology, Helios Klinikum, Siegburg, Germany, Siegburg, Nordrhein-Westfalen, Germany; Department of Pneumology, Infectiology, Internal Medicine and Intensive Care, Klinikum Dortmund GmbH, Dortmund, Dortmund, Nordrhein-Westfalen, Germany; Department of Gastroenterology, Hepatology and Infectiology, Heinrich-Heine-University, Duesseldorf, Germany, Düsseldorf, Nordrhein-Westfalen, Germany; Department of Internal Medicine I, University Medical Center of the Johannes Gutenberg University, Mainz, Germany, Mainz, Rheinland-Pfalz, Germany; Department of Internal Medicine II, Gastroenterology and Hepatology, Klinikum Worms, Germany, Worms, Rheinland-Pfalz, Germany; Department of Internal Medicine, Hematology/Oncology, Goethe University Frankfurt, Frankfurt am Main, Germany; University of Cologne, Faculty of Medicine and University Hospital Cologne, Department I of Internal Medicine, Center for Integrated Oncology Aachen Bonn Cologne Duesseldorf; German Centre for Infection Research (DZIF), partner site Bonn-Cologne, Cologne, Germany, Frankfurt am Main, Hessen, Germany

## Abstract

**Background:**

Numerous predictive clinical scores with varying discriminatory performance have been developed in the context of the current coronavirus disease 2019 (COVID-19) pandemic. To support clinical application, we test the transferability of the frequently applied 4C mortality score (4C score) to the German prospective Cross-Sectoral Platform (SUEP) of the National Pandemic Cohort Network (NAPKON) compared to the non COVID-19 specific quick sequential organ failure assessment score (qSOFA). Our project aims to externally validate these two scores, stratified for the most prevalent variants of concerns (VOCs) of severe acute respiratory syndrome coronavirus type 2 (SARS-CoV-2) in Germany.

**Methods:**

A total of 685 adults with polymerase chain reaction (PCR)-detected SARS-CoV-2 infection were included from NAPKON-SUEP. Patients were recruited from 11/2020 to 03/2022 at 34 university and non-university hospitals across Germany. Missing values were complemented using multiple imputation. Predictive performance for in-hospital mortality at day of baseline visit was determined by area under the curve (AUC) with 95%-confidence interval (CI) stratified by VOCs of SARS-CoV-2 (alpha, delta, omicron) (Figure 1).

**Figure 1:**
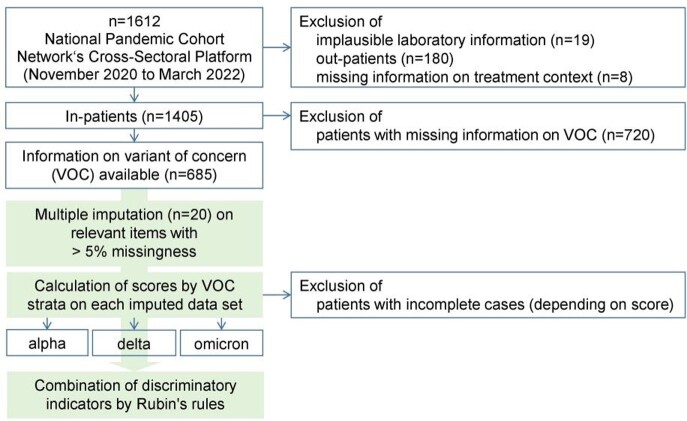
Study flow chart with inclusion criteria and methodological workflow.

**Results:**

Preliminary results suggest a high predictive performance of the 4C score for in-hospital mortality (Table 1). This applies for the overall cohort (AUC 0.813 (95%CI 0.738-0.888)) as well as the VOC-strata (alpha: AUC 0.859 (95%CI 0.748-0.970); delta: AUC 0.769 (95%CI 0.657-0.882); omicron: AUC 0.866 (95%CI 0.724-1.000)). The overall mortality rates across the defined 4C score risk groups are 0.3% (low), 3.2% (intermediate), 11.6% (high), and 49.5% (very high). The 4C score performs significantly better than the qSOFA (Chi^2^-test: p=0.001) and the qSOFA does not seem to be a suitable tool in this context.

**Table 1:**
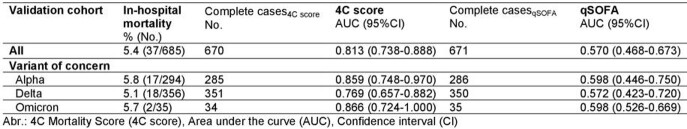
Discriminatory performance of the 4C Mortality Score and the qSOFA score within the validation cohort NAPKON-SUEP stratified by the Variant of Concerns of SARS-CoV-2.

**Conclusion:**

Despite its development in the early phase of the pandemic and improved treatment, external validation of the 4C score in NAPKON-SUEP indicates a high predictive performance for in-hospital mortality across all VOCs. However, since the qSOFA was not specifically designed for this predictive issue, it shows low discriminatory performance, as in other validation studies. Any interpretations regarding the omicron stratum are limited due to the sample size.

**Disclosures:**

**Daniel Pape, Dr.**, Advanz Pharma Germany: Support for attending meetings and/or travel for ECCMID 2021 **Martin Hower, n/a**, MSD: Advisor/Consultant|Trogarzo: Advisor/Consultant|ViiV Healthcare: Advisor/Consultant **Björn-Erik O. Jensen, Dr. med.**, GILEAD: Advisor/Consultant|GILEAD: Lectures, Travel|GSK: Lectures, Travel **Jörg J. Vehreschild, Univ.-Prof. Dr. med.**, Ärztekammer Nordrhein: Honoraria|Academy for Infectious Medicine, University Manchester: Honoraria|Astellas Pharma: Grant/Research Support|Astellas Pharma: Honoraria|Back Bay Strategies: Honoraria|Basilea: Grant/Research Support|Basilea: Honoraria|Deutsches Zetrum für Luft- und Raumfahrt (DLR): Grant/Research Support|German Centre for Infection Research (DZIF): Grant/Research Support|German Centre for Infection Research (DZIF): Honoraria|German Federal Ministry of Education and Research (BMBF): Grant/Research Support|German Society for Infectious Diseases (DGI): Honoraria|German Society for Internal Medicine (DGIM): Honoraria|GILEAD: Advisor/Consultant|GILEAD: Grant/Research Support|GILEAD: Honoraria|Janssen: Honoraria|Merck / MSD: Grant/Research Support|Merck / MSD: Honoraria|Molecular Health: Honoraria|Netzwerk Universitätsmedizin: Honoraria|NordForsk: Honoraria|Pfizer: Advisor/Consultant|Pfizer: Grant/Research Support|Pfizer: Honoraria|Rigshospitalet Copenhagen: Grant/Research Support|Shionogi: Advisor/Consultant|Shionogi: Honoraria|University Hospital Aachen: Honoraria|University Hospital Freiburg/ Congress and Communication: Honoraria|University of Bristol: Grant/Research Support.

